# Comparison between FOLFIRINOX and gemcitabine plus nab-paclitaxel including sequential treatment for metastatic pancreatic cancer: a propensity score matching approach

**DOI:** 10.1186/s12885-021-08277-7

**Published:** 2021-05-11

**Authors:** Jung Won Chun, Sang Hyub Lee, Joo Seong Kim, Namyoung Park, Gunn Huh, In Rae Cho, Woo Hyun Paik, Ji Kon Ryu, Yong-Tae Kim

**Affiliations:** 1 Center for Liver and Pancreatobiliary Cancer, Research Institute and Hospital, National Cancer Center, Goyang, South Korea; 2 Department of Internal Medicine and Liver Research Institute, Seoul National University Hospital, Seoul National University College of Medicine, Seoul, South Korea

**Keywords:** Pancreatic neoplasms, Folfirinox, Albumin-bound paclitaxel, Survival

## Abstract

**Background:**

FOLFIRINOX (FFX) and Gemcitabine plus nab-paclitaxel (GnP) have been recommended as the first-line chemotherapy for metastatic pancreatic cancer (mPC). However, the evidence is lacking comparing not only two regimens, but also sequential treatment (FFX–GnP vs. GnP–FFX).

**Methods:**

Data of 528 patients (FFX, *n* = 371; GnP, *n* = 157) with mPC were collected retrospectively. Propensity score matching was conducted to alleviate imbalance of the two groups. Overall survival (OS), progression free survival (PFS), and toxicity of patients were analyzed.

**Results:**

In the whole population, OS (12.5 months vs. 10.3 months, *P* = 0.05) and PFS (7.1 months vs. 5.8 months, *P* = 0.02) were longer in the FFX group before matching and after matching (OS: 11.8 months vs. 10.3 months, *P* = 0.02; PFS: 7.2 months vs. 5.8 months, *P* <  0.01). For sequential treatment, OS and PFS showed no significant difference. Interruptions of chemotherapy due to toxicities were more frequent (6.8 vs. 29.3%, *P* <  0.001) in the GnP group, and cessation of chemotherapy showed a significant association with mortality (z = − 1.94, *P* = 0.03).

**Conclusions:**

FFX achieved a longer overall survival than GnP in mPC, but not in the comparison for sequential treatment. More frequent adverse events followed by treatment interruptions during GnP might lead to a poor survival outcome. Therefore, FFX would be a better first-line treatment option than GnP for mPC.

**Supplementary Information:**

The online version contains supplementary material available at 10.1186/s12885-021-08277-7.

## Background

Pancreatic cancer (PC) is the seventh leading cause of cancer deaths worldwide. It is predicted to be the second-leading cause of cancer death in the USA by 2030 [1, 2]. Patients with PC have a poor prognosis, with a 5-year survival rate of 9%. Only less than 20% of patients with PC are resectable at initial diagnosis [2]. Most patients are diagnosed in an advanced stage. Chemotherapy is the mainstay treatment strategy for unresectable pancreatic cancer. However, options of chemotherapeutic regimen are still limited and some of them are often ineffective. Since single-agent gemcitabine was introduced as an effective treatment option in 1997, combination regimens are currently considered to be superior to monotherapy for advanced pancreatic cancer [3, 4]. It has been demonstrated that FOLFIRINOX (FFX, a combination of 5-fu, leucovorin, irinotecan, and oxaliplatin) and gemcitabine plus nab-paclitaxel (GnP) can significantly improve response rates and survival benefits compared to gemcitabine only in the randomized phase III PRODIGE and MPACT trials, respectively [5, 6]. Therefore, these two combination regimens are now recommended as first-line treatment for metastatic pancreatic cancer (mPC) [7–9].

There is no prospective study comparing FFX to GnP directly in mPC. Although the median survival of GnP is numerically shorter (8.5 months vs. 11.1 months) than FFX in those landmark trials, proportions of elderly patients, tumor burden, and patients with Eastern Cooperative Oncology Group performance status (ECOG-PS) 2 were higher in the MPACT trial [10]. Differences in enrolled population and study design make it difficult to directly compare their efficacy outcomes. In a real-world retrospective data of patients with advanced pancreatic cancer, two regimens showed comparable efficacy in terms of overall survival (OS) and progression free survival (PFS) [11]. And a recent meta-analysis including 16 retrospective or cohort studies also reported no major benefit of FFX in survival or response rate [12].

The survival benefit of FFX and GnP has been demonstrated and approximately half of patients with advanced pancreatic cancer receive second-line treatment [10]. Maintaining effective treatment for as long as possible is an important issue for patients with advanced pancreatic cancer which showed poor overall survival. Therefore, it is necessary to consider the subsequent chemotherapy when selecting the first-line chemotherapy regimen. In mPC, both FFX and GnP are available as second-line treatment option after progression on each exchanged regimen. However, the evidence for the effect of treatment sequence on OS is lacking. Therefore, the aim of this study was to compare the efficacy between FFX and GnP as well as their sequential treatment (FFX–GnP vs. GnP–FFX) in mPC in real world practice.

## Methods

### Study population

Patients who received FFX or GnP as first-line chemotherapy for mPC or recurrent PC after curative-intent resection at a tertiary university hospital in Korea between May 2011 and March 2019 were retrospectively reviewed in this study. Eligible patients were: (i) over 19 years old; (ii) pathologically confirmed pancreatic ductal adenocarcinoma; (iii) measurable disease according to the Response Evaluation Criteria in Solid Tumors, version 1.1; (iv) and ECOG-PS 2 or less. Patients who were lost to follow-up without initial tumor response evaluation were excluded. Data about patient’s age, sex, laboratory finding, tumor size, tumor location, and image findings were retrospectively collected from the electronic medical records system. The cut-off date for data analysis was June 30, 2020. Information on survival data was obtained from the Ministry of the Interior and Safety in Korea. This study was approved by the Institutional Review Board of Seoul National University Hospital (IRB No. C-2009-058-1155). It was conducted in accordance with the Declaration of Helsinki.

### Chemotherapy regimens and response evaluation

FFX was administered in combination with oxaliplatin (85 mg/m^2^), leucovorin (400 mg/m^2^), irinotecan (180 mg/m^2^), and 5-FU (400 mg/m^2^ bolus, 2400 mg/m^2^ continuous infusion) via intravenous route every 2 weeks [5]. GnP regimen consisting of gemcitabine (1000 mg/m^2^) and nab-paclitaxel (125 mg/m^2^) was administered on days 1, 8, and 15 every 4 weeks [6]. Dose reduction (by 10–30% of one or more drug) or schedule adjustment were performed in consideration of individual patient’s general condition and toxicities at the discretion of the clinician.

To evaluate treatment response, contrast enhanced computed tomography or magnetic resonance imaging was performed every 8 weeks. It was judged by the clinician according to the Response Evaluation Criteria in Solid Tumors. The detailed schedule of imaging studies was adjusted based on the patient’s symptoms and laboratory findings. Treatment-related adverse events (AEs) were evaluated and graded in accordance with the Common Terminology Criteria for Adverse Events, version 4.0. Chemotherapy-induced peripheral neuropathy was assessed based on the requirement for anti-neuropathic drugs (pregabalin, carbamazepine, and gabapentin) or chemotherapy dose reduction due to neuropathic symptoms.

### Objectives and statistical analyses

The primary objective was to compare OS and PFS between FFX and GnP according to their sequence. A propensity score matching (PSM) was used to alleviate the difference in baseline characteristics between the two groups of the study population. OS was defined as the time from the date of starting the first-line chemotherapy to the date of death or the last follow-up. PFS was defined as the time from the date of starting the first-line chemotherapy until the diagnosis of disease progression, death of any cause, or the last follow-up. PFS of sequential treatment was defined as the time between the first-line treatment and the second progression (progression after second-line treatment). Objective response rate (ORR) was defined as the percentage of patient with complete response or partial response. Disease control rate (DCR) was defined as ORR plus the rate of stable disease.

To estimate the propensity score, we used logistic regression including the following covariates: age, sex, ECOG-PS, number of metastatic sites, liver metastases, peritoneal seeding, lung metastases, previous curative pancreatic resection, location of primary tumor, and laboratory findings of baseline white blood cell counts and hemoglobin (Hb) levels. We matched the two groups of patients using a one-to-one nearest-neighbor matching protocol without replacement with a caliper width of 0.1 [13]. Standard mean differences were indicated before and after PSM.

Categorical data are expressed as count (percentage). Continuous data are expressed as median (interquartile range, IQR). Continuous variables were compared by independent student’s *t*-analysis. Non-continuous variables were compared by Chi-square or Fisher’s exact test. Median follow-up was calculated using the reverse Kaplan-Meier method [14]. Survival was estimated using the Kaplan-Meier method and compared by log-rank test. Factors affecting survival were determined by Cox proportional hazard regression model including as follows: age ≥ 65 years, sex, ECOG-PS ≥ 1, presence of number of metastatic sites ≥2, liver metastases, peritoneal seeding, and chemotherapy regimen identified as prognostic factors of survival in previous and present studies [15, 16]. A *P* value < 0.05 was considered statistically significant. All statistical analyses were performed using SAS systems version 9.2 (SAS Institute, Inc., Cary, NC, USA) and SPSS 24.0 statistical software (SPSS Inc., Chicago, IL, USA).

## Results

### Patient characteristics in the whole population and matched population

A total of 528 patients who were treated with FFX (371 patients) or GnP (157 patients) as the first-line chemotherapy were included in the final analysis. Baseline characteristics of these patients are presented in Table 1. Patients in the FFX group were significantly younger (61 years vs. 64 years, *P* <  0.001) than those in the GnP group. However, ECOG-PS was not significantly different (*P* = 0.15) between the two groups. Recurrent pancreatic cancer after curative surgery was more frequent in the GnP group (25.5% vs. 17.3%, *P* = 0.03). The median time to recurrence was 7.8 months (IQR: 4.0–18.3 months). There was no significant difference in tumor size, primary tumor location, or number of metastatic sites between the two groups. Peritoneal seeding (*P* = 0.03) and lung metastases (*P* = 0.02) were more common in the GnP group. The median Hb level was higher in the GnP group (12.5 g/dL vs. 12.2 g/dL, *P* = 0.03), although the numerical difference did not seem to be significant. After PSM, there were no significant difference in baseline characteristics (median age, level of Hb, proportion of previous curative surgery, peritoneal seeding, or lung metastases) between the two groups (Table 1).

**Table 1:** Baseline characteristics of the population before and after propensity score matching

	Whole study population				Matched population			
	FOLFIRINOX (*n* = 371)	Gemcitabine/nab-paclitaxel (*n* = 157)	*P* value	SMD	FOLFIRINOX (*n* = 151)	Gemcitabine/nab-paclitaxel (*n* = 151)	*P* value	SMD
Age (years)	61 (54–67)	64 (57–71)	< 0.001	0.38	62 (57–70)	64 (57–70)	0.39	0.05
Sex, male	229 (61.7)	94 (59.9)	0.69	0.04	84 (55.6)	91 (60.3)	0.42	− 0.09
ECOG-PS			0.15	−0.07			0.90	0.03
0	64 (17.3)	34 (21.7)			33 (21.9)	32 (21.2)		
1	305 (82.2)	120 (76.4)			116 (76.8)	116 (76.8)		
2	2 (0.5)	3 (1.9)			2 (1.3)	3 (2.0)		
Tumor size^a^	3.7 (3.0–4.8)	4.0 (2.6–5.3)	0.41	0.09	4.0 (3.0–4.8)	3.9 (2.5–5.0)	0.92	0.02
Tumor location^a^			0.07	−0.06			0.63	−0.01
Head/uncinate	115 (37.5)	33 (28.2)			37 (31.9)	33 (28.9)		
Body/tail	192 (62.5)	84 (72.4)			79 (68.1)	81 (71.1)		
Previous curative surgery	64 (17.3)	40 (25.5)	0.03	0.19	35 (23.2)	37 (24.5)	0.79	0.03
Number of metastatic sites			0.15	0.09			0.78	−0.07
1	233 (62.8)	97 (61.8)			88 (58.3)	94 (62.3)		
2	97 (26.1)	34 (21.7)			37 (24.5)	33 (21.9)		
3 or more	41 (11.1)	26 (16.6)			26 (17.2)	24 (15.9)		
Metastatic sites								
Liver metastases	234 (63.1)	90 (57.3)	0.20	−0.12	88 (58.3)	87 (57.6)	0.91	−0.01
Peritoneal seeding	100 (27.0)	57 (36.3)	0.03	0.19	53 (35.1)	53 (35.1)	1	0
Lung metastases	59 (15.9)	38 (24.2)	0.02	0.19	35 (23.2)	35 (23.2)	1	0
Distant lymph node	125 (33.7)	51 (32.5)	0.79	−0.03	51 (33.8)	49 (32.5)	0.81	−0.03
Other (bone, adrenal)	38 (10.2)	22 (14.0)	0.20	0.11	16 (10.6)	20 (13.2)	0.48	0.08
Laboratory findings								
WBC (cells/uL)	6.8 (5.6–8.7)	6.6 (5.1–8.4)	0.22	−0.09	6.8 (5.6–8.4)	6.6 (5.1–8.4)	0.687	−0.05
Hb (g/dL)	12.5 (11.3–13.7)	12.2 (11.1–13.1)	0.03	−0.19	12 (11–13)	12 (11–13)	0.583	0.09
PLT (× 103 cells/uL)	233 (185–286)	226 (182–287)	0.49	−0.04	235 (189–287)	224 (180–287)	0.338	−0.09
Bilirubin (mg/dL)	0.6 (0.4–0.9)	0.6 (0.5–0.8)	0.28	−0.07	0.6 (0.4–1.0)	0.6 (0.5–0.8)	0.873	−0.07
ALP (IU/L)	82 (68–146)	89 (72–123)	0.45	−0.06	88 (66–130)	89 (72–123)	0.874	−0.01
Albumin (g/dL)	3.9 (3.6–4.2)	4.0 (3.7–4.2)	0.50	0.02	3.9 (3.6–4.2)	4.0 (3.7–4.2)	0.195	0.09
CA 19–9 (U/mL)	1010 (121–5710)	938 (115–5320)	0.80	−0.54	676 (58–4560)	937 (115–5740)	0.481	−0.02

*FFX* folfirinox, *GnP* gemcitabine plus nab-paclitaxel, *SMD* standardized mean difference, *ECOG-PS* Eastern Cooperative Oncology Group performance status, *WBC* white blood cell count, *Hb* hemoglobin, *PLT* platelet count, *ALP* alkaline phosphatase, *CA* carbohydrate antigen

^a^Excluding previous curative resection cases

### Treatment efficacy of FOLFIRINOX and gemcitabine plus nab-paclitaxel

During the median follow-up period of 33 months, 83% patients (438 in the whole population; and 251 in the matched population) died after palliative chemotherapy. Detailed treatment data are presented in Table 2. In the first-line treatment setting, the median number of cycles of chemotherapy was 9 (IQR: 4–15) in the FFX group and 5 (IQR: 3–8) in the GnP group. Regardless of matching, the median treatment duration was significantly longer in the FFX group (5.6 months vs. 4.3 months, *P* = 0.03 in the whole population; 5.9 vs. 4.3 months, *P* = 0.01 in the matched population). In the FFX group, dose modification occurred more frequently (46% vs. 36%, *P* = 0.03 before matching; 50% vs. 34%, *P* = 0.01 after matching) during treatment, while interruption of chemotherapy (cessation or changing the regimen) was less frequent (7% vs. 29% in the whole population; 8% vs. 23% in the matched population; both *P* <  0.001) compared with the GnP group. Cessation chemotherapy among interruptions was significantly associated with more deaths (*P* = 0.03 by z-test; z = − 1.94).

**Table 2:** Detailed treatment data according to first-line chemotherapy regimen

	Whole study population			Matched population		
	FOLFIRINOX (*n* = 371)	Gemcitabine/nab-paclitaxel (*n* = 157)	*P* value	FOLFIRINOX (*n* = 151)	Gemcitabine/nab-paclitaxel (*n* = 151)	*P* value
N of cycle during first-line chemotherapy	9 (4–15)	5 (3–8)		9 (4–15)	5 (3–8)	–
Treatment duration of first-line (months)	5.6 (1.8–9.8)	4.3 (2.3–7.0)	0.03	5.9 (2.2–10.1)	4.3 (2.2–6.9)	0.01
Dose reduction during first-line chemotherapy	172 (46.4)	57 (36.3)^a^	0.03	76 (50.3)	52 (34.4)^a^	0.01
Interruption^b^ chemotherapy due to AEs	25 (6.8)	46 (29.3)	< 0.001	12 (7.9)	35 (23.1)	< 0.001
Cessation treatment	21 (5.7)	24 (15.3)		11 (7.3)	28 (18.5)	
Change regimen	4 (1.1)	22 (14.0)		1 (0.7)	7 (4.6)	
Best response^c^			0.33			0.29
CR	4 (1.1)	0 (0)	0.18	3 (2.0)	0 (0)	
PR	106 (28.9)	48 (32.4)		48 (32.0)	46 (32.4)	
SD	166 (45.2)	71 (48.0)		64 (42.7)	68 (47.9)	
PD	91 (24.8)	29 (19.6)		35 (23.3)	28 (19.7)	
Response rates^c^						
ORR	110 (30.0)	48 (32.4)	0.58	51 (34.0)	46 (32.4)	0.77
DCR	276 (75.2)	119 (80.4)	0.21	115 (76.7)	114 (80.3)	0.45
Second-line treatment	245 (69.2)	92 (58.6)	0.02	103 (71.5)	88 (58.3)	0.02
FOLFIRINOX	–	61		–	59	
Nab-paclitaxel	120	–		53	–	
Clinical trial	33	5		14	4	
Other Gemcitabine^d^	86	0		34	0	
Other 5-FU^e^	6	26		2	25	

*FFX* FOLFIRINOX, *GnP* gemcitabine plus nab-paclitaxel, *AEs* adverse events, *CR* complete response, *PR* partial response, *SD* stable disease, *PD* progression of disease, *ORR* objective response rate, *DCR* disease control rate, *5-FU* 5-fluorouracil, *FOLFOX* oxaliplatin plus 5-FU/leucovorin, *FOLFIRI* irinotecan plus 5-FU/leucovorin, *iFAM* infusional 5-FU plus doxorubicin and mitomycin-C

^a^Included patients for whom nab-paclitaxel was omitted due to adverse events

^b^Discontinuation chemotherapy or switching of chemotherapy regimen

^c^Not evaluated for patients with regimen change or death before response evaluation

^d^Included gemcitabine single, gemcitabine plus erlotinib regimens

^e^Included FL, FOLFOX, FOLFIRI, iFAM, capecitabine plus oxaliplatin, tegafur/gimestat/otastat potassium regimens

**Fig. 1:**
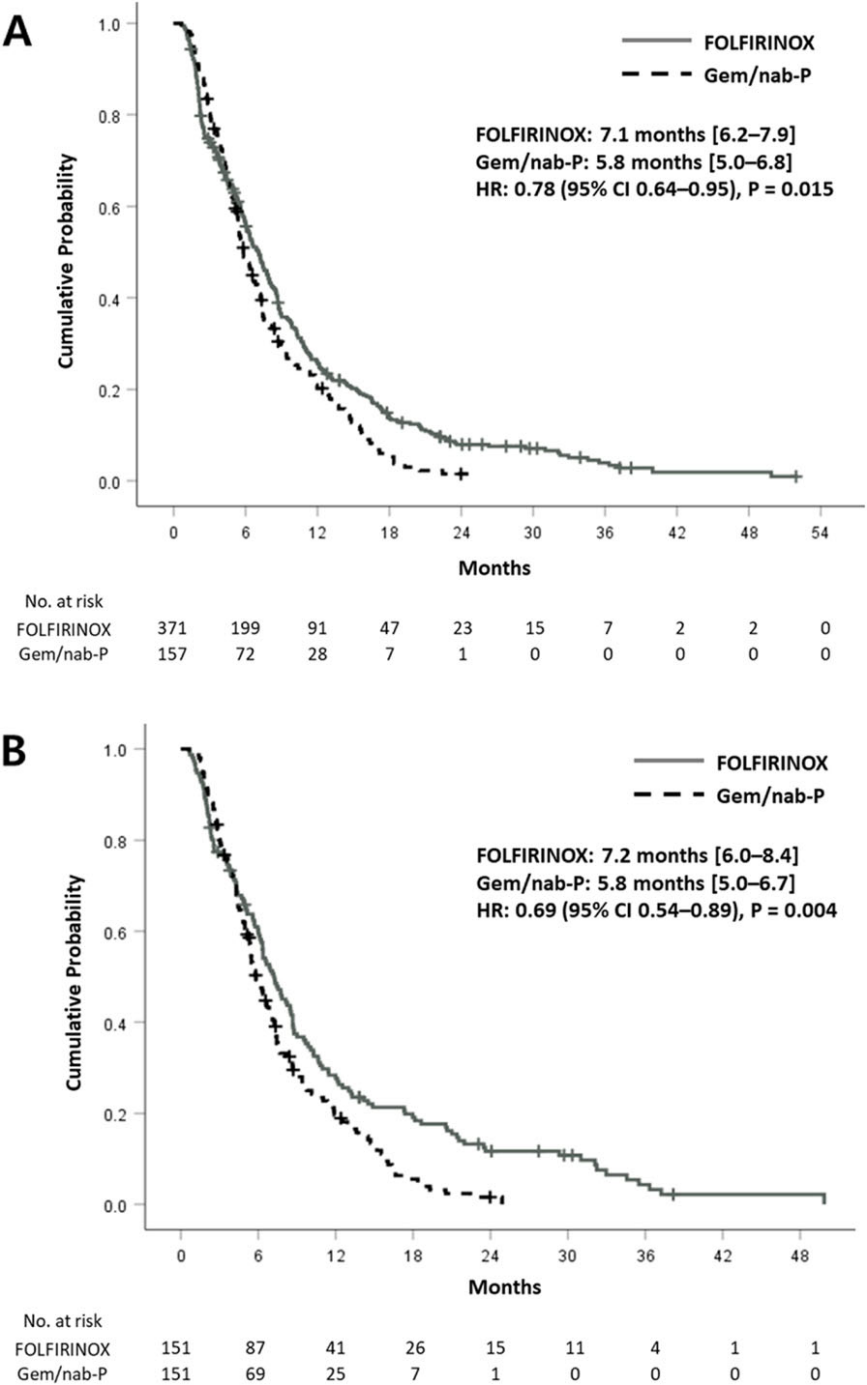
Progression free survival **a** before and **b** after propensity score matching

**Fig. 2:**
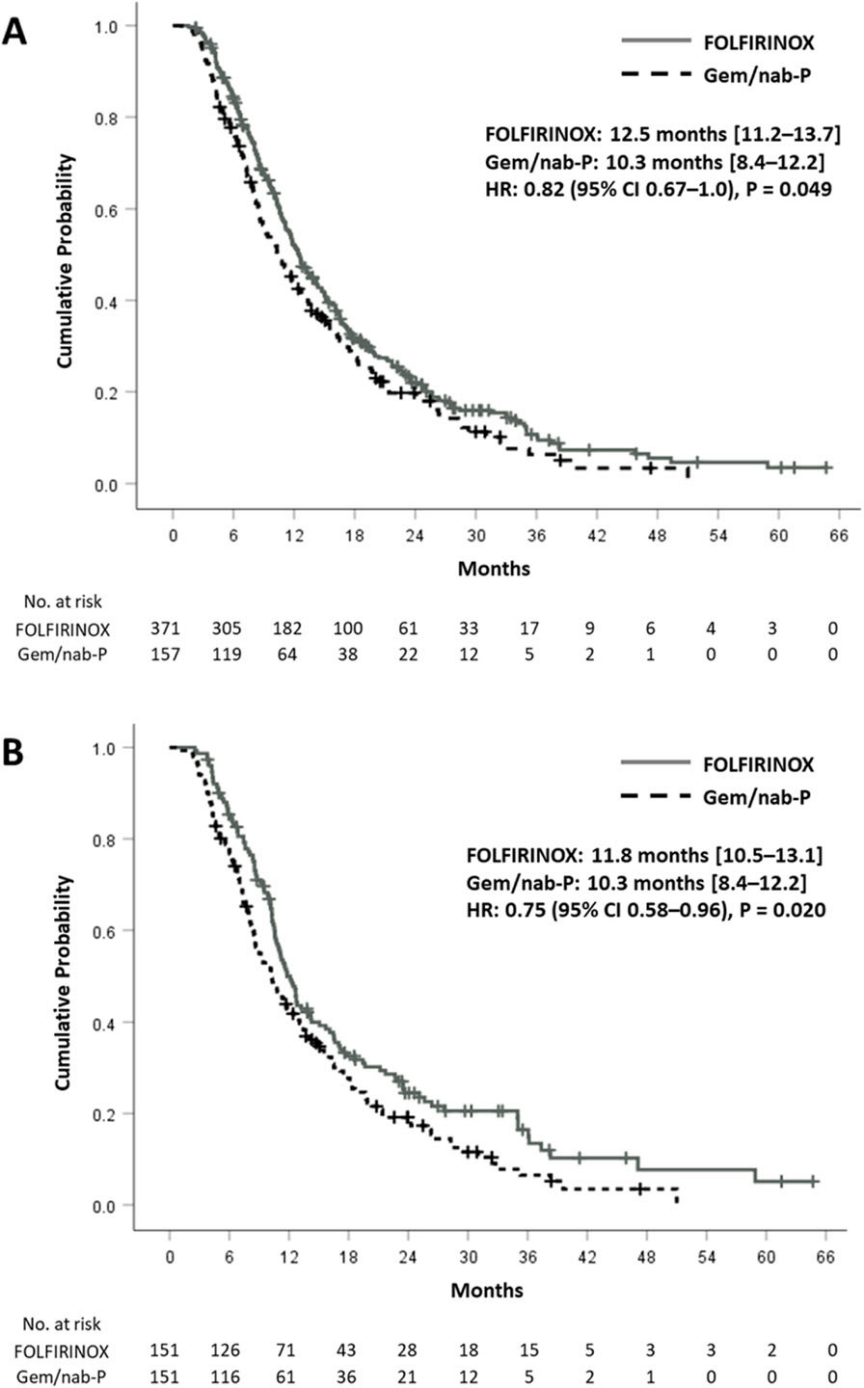
Overall survival **a** before and **b** after propensity score matching

In terms of treatment efficacy, response rates were not significantly different between the two groups before PSM (30% vs. 32%, *P* = 0.58) or after PSM (34% vs. 32%, *P* = 0.77). The median PFS was significantly longer in the FFX group both before PSM (7.1 months vs. 5.8 months, *P* = 0.02) and after PSM (7.2 months vs. 5.8 months, *P* <  0.01) compared to that in the GnP group (Fig. 1). In the whole population, FFX regimen showed a marginal benefit in the OS (12.5 months vs. 10.3 months, *P* = 0.05) (Fig. 2). Statistical significance of survival benefit was further guaranteed after PSM (11.8 vs. 10.3 months, *P* = 0.02). In multivariable adjusted analyses, survival benefit of FFX was supported, with hazard ratio (HR) of 0.80 (95% confidence interval [CI]: 0.65–0.98; *P* = 0.03) before matching and HR of 0.74 (95% CI: 0.57–0.95; *P* = 0.02) after matching (Supplementary Table 1). Additionally, age above 65 years, liver metastases, and peritoneal seeding were independently associated with poor survival. In subgroup survival analyses, patients with younger age (< 65 years), ECOG-PS 1+, and without history of previous pancreatic surgery showed statistically significant survival benefits with the FFX treatment (Supplementary Fig. 1–4).

### Sequential treatments of FOLFIRINOX (FFX–GnP) and gemcitabine plus nab-paclitaxel (GnP–FFX)

**Fig. 3:**
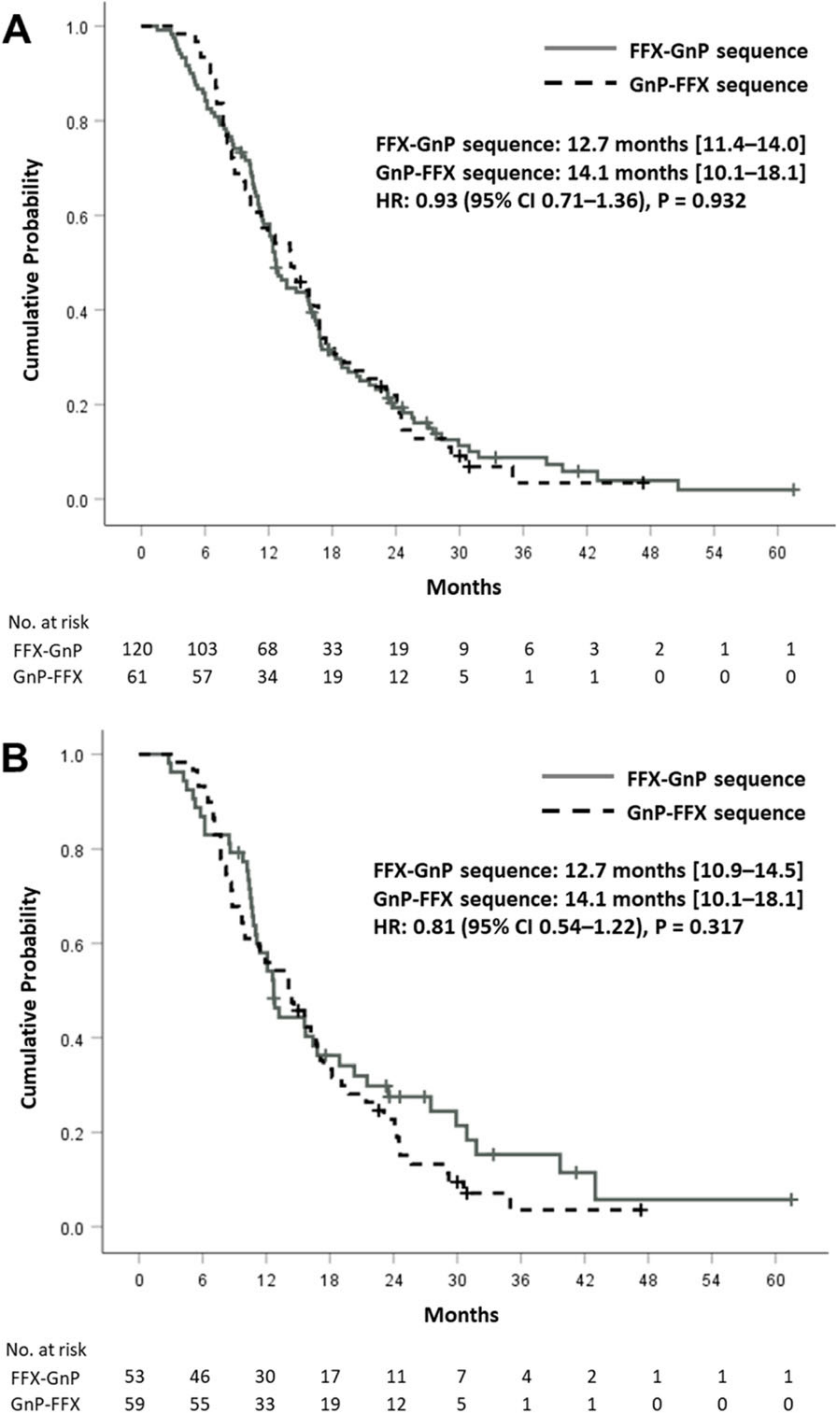
Progression free survival of sequential treatment in the **a** whole and **b** matched populations

**Fig. 4:**
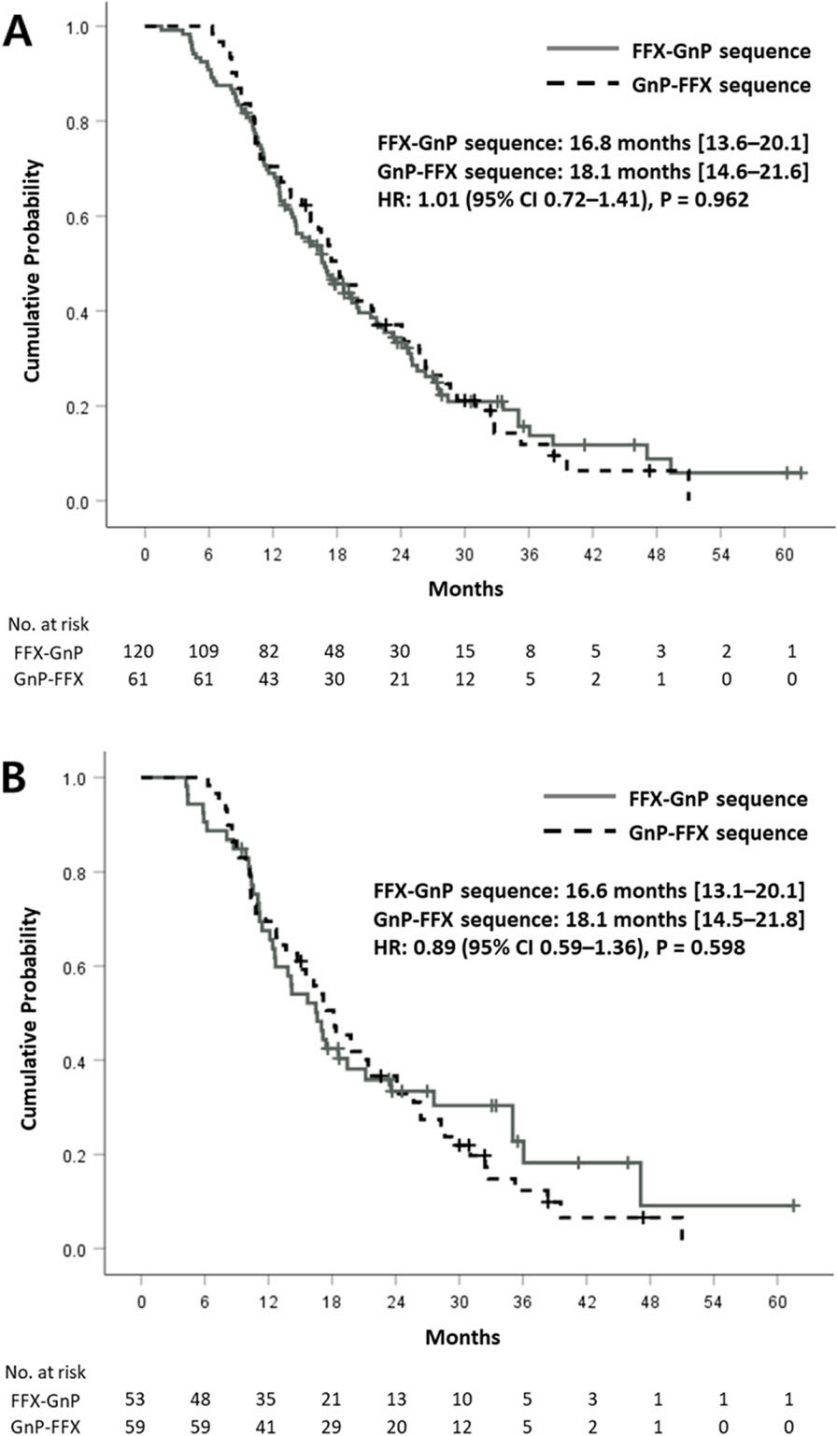
Overall survival of sequential treatment in the **a** whole and **b** matched populations

Regimens of second-line chemotherapy are summarized in Table 2. The proportion of patients who received subsequent chemotherapy beyond first-line therapy was significantly higher in the FFX group than in the GnP group (69% vs. 59% before matching; 72% vs. 58% after matching; both *P* = 0.02). The proportion of FFX–GnP sequence and reverse sequence (GnP–FFX) were not significantly different between the two groups. Treatment outcomes of sequential chemotherapy are presented in Table 3. There was no significant difference in ORR or DCR during second-line treatment. The rate of patients who underwent the third-line chemotherapy was not different between the two sequences. Regarding treatment efficacy, the median PFS was 12.7 months for the FFX–GnP sequence and 14.1 months for the GnP–FFX sequence without showing significant difference between the two sequences (Fig. 3). The median OS was not significantly different according to the sequence either (Fig. 4). There was no significant difference in survival according to the sequence of treatment for several subgroups (age, ECOG-PS, and previous curative surgery) (Supplementary Fig. 5–7).

**Table 3:** Treatment outcomes in patients with each sequential chemotherapy

	FFX-GnP (*n* = 120)	GnP-FFX (*n* = 61)	*P* value	FFX-GnP (*n* = 53)	GnP-FFX (*n* = 59)	*P* value
Treatment duration (months)	12.0 (7.6–18.1)	11.7 (7.6–19.4)	0.945	12.0 (8.0–22.3)	11.7 (7.4–20.0)	0.714
Best response in second-line treatment			0.86			0.69
CR	1 (0.8)	0 (0)		1 (1.9)	0 (0)	
PR	19 (15.8)	10 (16.4)		7 (13.2)	10 (16.9)	
SD	61 (50.8)	29 (47.5)		27 (50.9)	28 (47.5)	
PD	39 (32.5)	22 (36.1)		18 (34.0)	21 (35.6)	
Response rates in second-line treatment						
DCR	81 (67.5)	39 (63.9)	0.631	35 (66.0)	38 (64.4)	0.86
ORR	20 (16.7)	10 (16.4)	0.963	8 (15.1)	10 (16.9)	0.79
Third-line chemotherapy	47 (39.2)	29 (47.5)	0.508	16 (30.2)	28 (47.5)	0.14
Other 5-FU^a^	46	6		1	6	
Other gemcitabine^b^	1	21		15	20	
Other (clinical trial, SBRT)	0	2		0	2	

*FFX* FOLFIRINOX, *GnP* gemcitabine plus nab-paclitaxel, *AEs* adverse events, *CR* complete response, *PR* partial response, *SD* stable disease, *PD* progression of disease, *ORR* objective response rate, *DCR* disease control rate, *5-FU* 5-fluorouracil, *SBRT* stereotactic body radiotherapy, *NAPOLI* nanoliposomal irinotecan plus 5-FU/lecovorin, *iFAM* infusional 5-FU plus doxorubicin and mitomycin-C

^a^Included the NAPOLI, iFAM, tegafur/gimestat/otastat potassium, capecitabine, and CCRT with 5-FU or capecitabine regimens

^b^Included gemcitabine, gemcitabine plus erlotinib

### Treatment-related adverse events in the whole population and the matched population

Treatment-related AEs in the whole population and the matched population are shown in Table 4. Regarding hematologic toxicities, the GnP group had higher incidence of severe anemia (grade 3 to 4) before and after matching. The rate of febrile neutropenia occurred more frequently in the FFX group before matching (12.9% vs. 6.4%, *P* = 0.03), but not significantly different after matching (*P* = 0.30). Prophylactic granulocyte colony-stimulating factor (G-CSF) was more frequently used in the FFX group (*P* <  0.001). With regard to peripheral neuropathy, more patients in the GnP group needed anti-neuropathic drugs or dose reduction to relieve their symptoms with significant difference before matching (*P* <  0.01), but nonsignificantly different (*P* = 0.07) after matching.

**Table 4:** Treatment-related adverse events and causes of chemotherapy interruptions

	FOLFIRINOX (*n* = 371)	Gemcitabine/nab-paclitaxel (*n* = 157)	*P* value	FOLFIRINOX (*n* = 151)	Gemcitabine/nab-paclitaxel (*n* = 151)	*P* value
Treatment-related adverse events						
Anemia ≥3	70 (18.9)	50 (31.8)	< 0.01	29 (19.2)	50 (33.1)	0.01
Thrombocytopenia ≥3	48 (12.9)	15 (9.6)	0.27	22 (14.6)	15 (9.9)	0.22
Neutropenia ≥3	181 (48.8)	70 (44.6)	0.38	78 (51.7)	67 (44.4)	0.21
Neutropenic fever	48 (12.9)	10 (6.4)	0.03	15 (9.9)	10 (6.6)	0.30
Granulocyte colony stimulating factor	304 (81.9)	10 (6.4)	< 0.001	130 (86.1)	9 (6.0)	< 0.001
Peripheral neuropathy	56 (15.1)	40 (25.5)	< 0.01	25 (16.6)	38 (25.2)	0.07
Cause of chemotherapy interruption	25 (6.7)	46 (29.3)	< 0.001	12 (7.9)	35 (23.2)	< 0.001
General weakness	21 (5.6)	22 (14.0)		10 (6.6)	19 (12.6)	
Peripheral neuropathy	0	15 (9.6)		0	8 (5.3)	
Peripheral edema	0	3 (1.9)		0	3 (2.0)	
Uncontrolled diarrhea	1 (0.3)	1 (0.6)		0	1 (0.7)	
Drug-induced pneumonitis	0	2 (1.3)		0	1 (0.7)	
Bone marrow suppression	3 (0.8)	1 (0.6)		2 (1.3)	1 (0.7)	
Others (pneumonia, tumor bleeding)	0	2 (1.3)		0	2 (1.4)	

Causes of interruption chemotherapy due to AEs are listed in Table 4. Interruptions occurred significantly more in the GnP group as described above. General weakness (deterioration of performance) was the most common cause of in both groups, whereas peripheral neuropathy was another notable cause in the GnP group. Drug-induced pneumonitis and peripheral edema were observed only in patients of the GnP group.

## Discussion

Current guidelines recommend FFX and GnP as first-line treatment regimens of mPC [7–9]. The present study showed an overall survival of 12 months in the FFX group and 10 months in the GnP group, in agreement with respective pivotal trials [5, 6]. Statistical significance of survival in the FFX group was preserved even after PSM. In subgroups of patients with younger age, ECOG-PS 1+, and without a history of curative surgery, FFX treatment showed statistically significant survival benefits. With sequential treatment, the median OS was comparable between FFX–GnP (17 months) and GnP–FFX (18 months) sequences in the whole population and the matched population. Regarding treatment-related AEs, febrile neutropenia occurred more frequently in the whole population, but not significantly different between the two groups in the matched population, whereas severe anemia and peripheral neuropathy occurred more frequently in the GnP group. Interruptions of chemotherapy due to AEs occurred more frequently in patients who received GnP. Major causes of interruptions were performance deterioration and peripheral neuropathy.

A recent systematic review has compared efficacies of FFX and GnP as first-line treatment in mPC patients and found no difference in efficacy regarding the risk of death and disease progression between the two regimens despite a numerical difference in OS (mean difference: 1.15; *P* = 0.03) in favor of FFX [12]. And three recent published retrospective studies have also compared FFX and GnP in mPC patients (Supplementary Table 2) [17–19]. Among these, two studies have shown superior OS for patients treated with FFX [17, 19]. They performed propensity score analysis to reduce the bias due to confounding variables such as age, sex, ECOS-PS, and so on known to be import prognostic factors of survival [16]. We also performed PSM to lower differences in baselines between the two groups. After PSM, the significance of survival benefit by FFX as first-line chemotherapy was statistically reinforced (from *P* = 0.05 to *P* = 0.02). Regarding response rate, ORR and DCR of the GnP were not significantly different from those of the FFX group as first- and second-line treatments including their sequential treatment.

FFX is usually preferred for younger patients in good performance status [12]. In this study, FFX also showed favorable survival in the subgroup of patients under age 65. However, OS in patients with ECOG-PS 0 showed no significant different between the two regimens. ECOG-PS did not show prognostic significance for OS in the proportional hazards model. Rather, cessation chemotherapy among interruptions was significantly associated with poor survival. The proportion of interruption of chemotherapy due to AEs was significantly higher in the GnP regimen. The main cause of interruption was deterioration of performance status. A previous study has also reported that the rate of cessation chemotherapy due to AEs was (nonsignificantly) higher in the GnP group (14% vs. 21%) with general weakness being the main cause of early-termination of chemotherapy [18]. These interruptions might account for the result of a shorter treatment duration in the GnP group in this study despite no difference in follow-up period (33 months in the FFX group vs. 32 months in the GnP group, *P* = 0.91). It was confirmed that the median OS was longer in the patients with FFX who had interruptions, especially cessation of chemotherapy (Supplementary Fig. 8–9).

Performance status decline due to GnP could also explain the result that FFX treatment rather reduced the risk of death in patients with ECOG-PS 1+ in the subgroup analysis. In this subgroup, the incidence rate of cessation chemotherapy was much higher (17.9% vs. 5.5%, *P* <  0.001) in the GnP group compared to the result of the whole population. In another aspect of tolerability, dose modification was more frequently in FFX group. The efficacy and tolerability of FFX with modified dose for unresectable PC demonstrated in several studies [20, 21]. Modified FFX showed comparable survival outcomes compared to full dose regimen and less adverse events [20]. Appropriate dose modification and tolerance to chemotherapy would provide patients with more chances for second-line treatment, and more patients in the FFX group received second-line chemotherapy in this study.

Peripheral neuropathy is another notable cause of interruption chemotherapy in this study. Chemotherapy-induced peripheral neuropathy could be experienced by patients receiving both GnP (due to nab-paclitaxel) and FFX (due to oxaliplatin) regimens. In our results, however, significantly more patients suffered from neuropathy during GnP treatment and regimen change was more needed even if chemotherapy kept the tumor under control. In this regard the high probability of neuropathy development should be carefully taken into account when determining treatment with GnP. Further study is needed to reveal factors associated with the occurrence of GnP-induced neuropathy. Another concerned AE of chemotherapy was bone marrow suppression. Although neutropenia and febrile neutropenia are well-known AEs of FFX, there was no significant difference in the incidence of neutropenia or febrile neutropenia in the matched population in our results. This might be related to the predominantly higher use of prophylactic G-CSF in the FFX group. A similar result has been shown in a recent retrospective comparative study in Korea [18]. Conversely, another result of marrow suppression, severe anemia, was more frequent in the GnP group, in accordance with previous meta-analysis results [12]. Different patterns and severity of AEs between two regimens showed in this study, but the mechanism of theses difference was not elucidated. Translational research, for example genome- or transcriptome-based comparison, would illuminate the factors associated with susceptibility to AEs, and targeting strategies could be used in clinical applications to reduce interruptions of chemotherapy and improve survival outcomes [22, 23].

With improved survival of pancreatic cancer in the last few decades, treatment sequences also became a factor to consider in chemotherapy. In the present data, both PFS and OS were comparable between FFX–GnP and GnP–FFX sequences. There was no significant difference in subgroup survival analyses. Response rates were not significantly different across each line of treatment in two sequences. Several studies have compared OS between two subsequent treatments and found no significant difference in survival benefit [17, 19, 24]. Williet et al. have assessed the feasibility of two sequences through the proportion of each sequence (43% in FFX–GnP vs. 13% in GnP–FFX) [17]. Although the rate of second-line FFX after GnP (32.3%) was comparable to that of GnP after FFX (38.9%) in our study, the proportion of patients who received second-line treatment was significantly higher in the FFX first group. Therefore, when considering the efficacy, feasibility, and AEs (especially, deterioration of performance and peripheral neuropathy), FFX as the first-line treatment could be a better choice of chemotherapeutic regimen.

The main limitation of this study came from its retrospective design. Hence, exact data could not be obtained. We performed PSM with a relatively large number of patients to alleviate potential confounders. Standardized mean differences were lesser than 10% after matching which confirmed that our data were well-balanced [13]. The absence of comparing the cost-effectiveness analysis of two regimens was another limitation. Cho et al. have shown that costs of these two regimens are not very different for first-line setting [18]. However, second-line FFX or GnP is not covered under national health insurance system in Korea. This should be considered in determining the sequential treatment. Randomized controlled trials are warranted to clarify which regimen or sequence of the two regimens would be beneficial to patients with mPC. Our results showed the survival benefit of FFX first treatment, and the main explanation is different tolerance to chemotherapy. However, the biological mechanism was not elucidated, and the research using preclinical models of pancreatic cancer is needed to illuminate the biomolecular meaning of clinical findings.

One of the strengths of our study was that it had a relatively large number of patients compared to previous retrospective studies, even after PSM. Patient matching was well-performed with standardized mean differences under 10% and the survival benefit of FFX was maintained after matching. Therefore, this study would provide valid comparative results of two regimens and sequences in the absence of a randomized comparative study. We also suggested a possible explanation of survival benefit of FFX fist regimen, less interruptions of treatment (mainly cessation chemotherapy) due to AEs. However, if the reverse sequence can be performed through second-line therapy, there will be no significant difference in survival.

In conclusion, results of our study support the survival benefit of FFX as first-line treatment for mPC. Our results also showed comparable efficacy between two sequences. FFX first sequence would be tolerable in terms of a lower rate of interrupting chemotherapy due to AEs than GnP. However, even in cases of treatment with GnP as first-line regimen, second-line FFX would ensure comparable survival outcome in patients with adequate performance. Further studies are warranted to identify patients who are fit for each regimen and sequence. Further studies are also needed to illuminate risk factors for chemotherapy-related AEs in order to screen chemotherapy interruption due to AEs.

## Supplementary Information

**Additional file 1: Supplementary Table 1**. Uni−/multi-variable analyses of prognostic factors for overall survival. **Supplementary Table 2**. Recently published studies comparing FOLFIRINOX vs Gemcitabine/nab-paclitaxel including sequential treatment. **Supplementary Figure 1**. Subgroup analyses for overall survival in the (A) whole and (B) matched populations according to the first-line treatment regimens. **Supplementary Figure 2**. Kaplan–Meier curves of overall survival among patients with age < 65 years in the (A) whole and (B) matched populations. **Supplementary Figure 3**. Kaplan–Meier curves of overall survival among patients with ECOG 1+ in the (A) whole and (B) matched populations. **Supplementary Figure 4**. Kaplan–Meier curves of overall survival among patients without previous pancreatic resection in the (A) whole and (B) matched populations. **Supplementary Figure 5**. Kaplan–Meier curves of overall survival in patients with sequential treatment according to patients’ age. (A) Age < 65 years; (B) Age ≥ 65 years. **Supplementary Figure 6**. Kaplan–Meier curves of overall survival in patients with sequential treatment according to performance status. (A) ECOG-PS 0; (B) ECOG-PS 1. **Supplementary Figure 7**. Kaplan–Meier curves of overall survival in patients with sequential treatment according to history of pancreatic resection. (A) Naïve status; (B) With resection. **Supplementary Figure 8**. Kaplan–Meier curves of overall survival in patients with interruptions of chemotherapy (A) before matching and (B) after matching. **Supplementary Figure 9**. Kaplan–Meier curves of overall survival in patients with cessation of chemotherapy (A) before matching and (B) after matching

## Data Availability

The datasets used and/or analyzed during the current study are available from the corresponding author (Sang Hyub Lee) on reasonable request.

## Abbreviations

PC: Pancreatic cancer
FFX: FOLFIRINOX
GnP: Gemcitabine plus nab-paclitaxel
mPC: Metastatic pancreatic cancer
ECOG-PS: Eastern Cooperative Oncology Group performance status
OS: Overall survival
PFS: Progression survival
FFX–GnP: FOLFIRINOX followed by sequential gemcitabine plus nab-paclitaxel
GnP–FFX: Gemcitabine plus nab-paclitaxel followed by sequential FOLFIRINOX
AE: Adverse event
PSM: Propensity score matching
ORR: Objective response rate
DCR: Disease control rate
Hb: Hemoglobin
IQR: Interquartile range
HR: Hazard ratio
CI: Confidence interval
