# Supplementary material for: Comparison between FOLFIRINOX and gemcitabine plus nab-paclitaxel including sequential treatment for metastatic pancreatic cancer: a propensity score matching approach
Source: BMC Cancer. 2021 May 11;21:537. doi: 10.1186/s12885-021-08277-7 (PMC8114681; doi:10.1186/s12885-021-08277-7)
Supplement: Supplementary file 1 — Additional file 1: Supplementary Table 1. Uni−/multi-variable analyses of prognostic factors for overall survival. Supplementary Table 2. Recently published studies comparing FOLFIRINOX vs Gemcitabine/nab-paclitaxel including sequential treatment. Supplementary Figure 1. Subgroup analyses for overall survival in the (A) whole and (B) matched populations according to the first-line treatment regimens. Supplementary Figure 2. Kaplan–Meier curves of overall survival among patients with age < 65 years in the (A) whole and (B) matched populations. Supplementary Figure 3. Kaplan–Meier curves of overall survival among patients with ECOG 1+ in the (A) whole and (B) matched populations. Supplementary Figure 4. Kaplan–Meier curves of overall survival among patients without previous pancreatic resection in the (A) whole and (B) matched populations. Supplementary Figure 5. Kaplan–Meier curves of overall survival in patients with sequential treatment according to patients’ age. (A) Age < 65 years; (B) Age ≥ 65 years. Supplementary Figure 6. Kaplan–Meier curves of overall survival in patients with sequential treatment according to performance status. (A) ECOG-PS 0; (B) ECOG-PS 1. Supplementary Figure 7. Kaplan–Meier curves of overall survival in patients with sequential treatment according to history of pancreatic resection. (A) Naïve status; (B) With resection. Supplementary Figure 8. Kaplan–Meier curves of overall survival in patients with interruptions of chemotherapy (A) before matching and (B) after matching. Supplementary Figure 9. Kaplan–Meier curves of overall survival in patients with cessation of chemotherapy (A) before matching and (B) after matching [file 12885_2021_8277_MOESM1_ESM.docx]

**Supplementary Material**

**Title: Comparison between FOLFIRINOX and Gemcitabine Plus Nab-paclitaxel Including Sequential Treatment for Metastatic Pancreatic Cancer: A Propensity Score-matching Approach**

Jung Won Chun^1^, Sang Hyub Lee^2^, Joo Seong Kim^2^, Namyoung Park^2^, Gunn Huh^2^, In Rae Cho^2^, Woo Hyun Paik^2^, Ji Kon Ryu^2^, Yong-Tae Kim^2^

^1^Center for Liver and Pancreatobiliary Cancer, Research Institute and Hospital, National Cancer Center, Goyang, Korea

^2^Department of Internal Medicine and Liver Research Institute, Seoul National University Hospital, Seoul National University College of Medicine, Seoul, Korea

Correspondence to: Sang Hyub Lee, MD, PhD

Professor, Department of Internal Medicine and Liver Research Institute, Seoul National University Hospital, Seoul National University College of Medicine

101 Daehak-ro, Jongno-gu, Seoul, 110-744, Korea

Tel: 82-2-2072-4892/Fax: 82-2-762-9662

Supplementary Table 1. Uni-/multi-variable analyses of prognostic factors for overall survival

|  | Whole population (n = 528) | | | | | Matched population (n = 302) | | | |
| --- | --- | --- | --- | --- | --- | --- | --- | --- | --- |
|  | Univariable | | Multivariable* | | Univariable | | | Multivariable* | |
|  | HR (95% CI) | P value | HR (95% CI) | P value | HR (95% CI) | | P value | HR (95% CI) | P value |
| Age ≥ 65 | 1.14 (0.94–1.39) | 0.183 | 1.22 (1.00–1.49) | 0.046 | 1.22 (0.95–1.57) | | 0.119 | 1.35 (1.05–1.74) | 0.02 |
| Sex, female | 0.87 (0.72–1.06) | 0.172 |  |  | 0.88 (0.68–1.13) | | 0.326 |  |  |
| ECOG-PS ≥ 1 | 1.08 (0.85–1.36) | 0.533 |  |  | 1.20 (0.90–1.60) | | 0.225 |  |  |
| Previous curative surgery | 0.85 (0.67–1.07) | 0.168 |  |  | 0.86 (0.64–1.15) | | 0.298 |  |  |
| N of metastatic sites ≥ 2 | 1.30 (1.07–1.58) | 0.008 |  |  | 1.52 (1.18–1.96) | | 0.001 |  |  |
| Liver metastases | 1.51 (1.24–1.84) | <0.001 | 1.77 (1.43–2.19) | <0.001 | 1.49 (1.15–1.92) | | 0.002 | 1.86 (1.41–2.45) | <0.001 |
| Peritoneal seeding | 1.17 (0.96–1.43) | 0.129 | 1.42 (1.14–1.76) | 0.002 | 1.38 (1.07–1.78) | | 0.014 | 1.83 (1.38–2.42) | <0.001 |
| Lung metastases | 0.96 (0.75–1.23) | 0.751 |  |  | 0.95 (0.70–1.28) | | 0.719 |  |  |
| Distant lymph node | 0.86 (0.70–1.05) | 0.144 |  |  | 1.05 (0.81–1.37) | | 0.702 |  |  |
| CA19-9 ≥ 800 U/mL | 1.15 (0.94–1.42) | 0.179 |  |  | 1.18 (0.90–1.55) | | 0.227 |  |  |
| FOLFIRINOX as first-line chemotherapy | 0.82 (0.67–1.00) | 0.049 | 0.80 (0.65–0.98) | 0.029 | 0.75 (0.58–0.96) | | 0.020 | 0.74 (0.57–0.95) | 0.016 |

HR, hazard ratio; ECOG-PS, Eastern Cooperative Oncology Group performance status; GnP, gemcitabine plus nab-paclitaxel

* Multivariable analyses were performed by forward LR method including age, sex, ECOG-PS, presence of number of metastatic site, liver metastases, peritoneal seeding, and initial chemotherapy regimen.

Supplementary Table 2. Recently published studies comparing FOLFIRINOX vs Gemcitabine/nab-paclitaxel including sequential treatment

| Author | Year | Study period | Country | Type of study,  Matching | Sample size | Age  (median) | Overall survival | Overall survival in sequential treated patients |
| --- | --- | --- | --- | --- | --- | --- | --- | --- |
| N Williet | 2019 | 2015-2018 | France | Retrosepctive,  PSM | 107 vs 109 before PSM  49 vs 49 after PSM | 62 vs 68 | 14 vs 9 months (P = 0.008) before PSM  14 vs 9 months (P = 0.097) after PSM | 21 vs 11 months (p = 0.11) before PSM  19 vs 9.5 months (p = 0.094) after PSM |
| Chan | 2020 | 2015-2017 | Canada | Retrosepctive,  IPTW | 216 vs 118 | 62 vs 70 | 8.2 vs 6.1 months (p < 0.0001) | 10.4 vs 8.4 months  FFX-gem (73.6%), -GnP (26.4%)  GnP-gem (90.0%), -FFX (10%) |
| Cho | 2020 | since 2015 | Korea | Retrosepctive,  none | 86 vs 81 | 54 vs 65 | 10.7 vs 12.1 (p = 0.157) | - |

PSM, propensity score matching; IPTW, Inverse probability of treatment weighting; FFX, FOLFIRINOX; GnP; Gemcitabine plus nab-paclitaxel

Supplementary Figure 1. Subgroup analyses for overall survival in the (A) whole and (B) matched populations according to the first-line treatment regimens.

| A   | B   |
| --- | --- |

Supplementary Figure 2. Kaplan–Meier curves of overall survival among patients with age < 65 years in the (A) whole and (B) matched populations.

| A  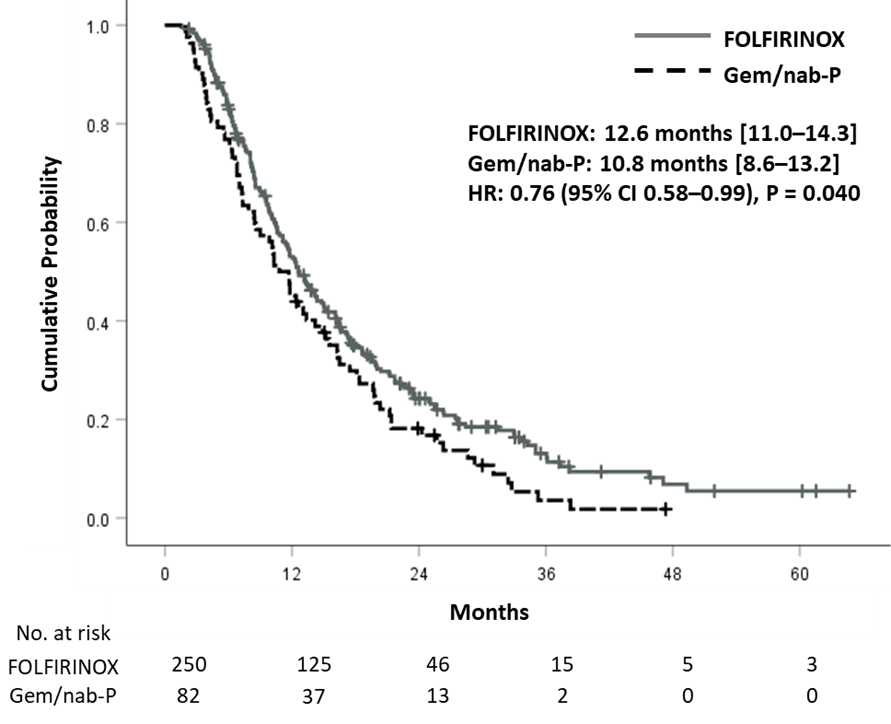 | B  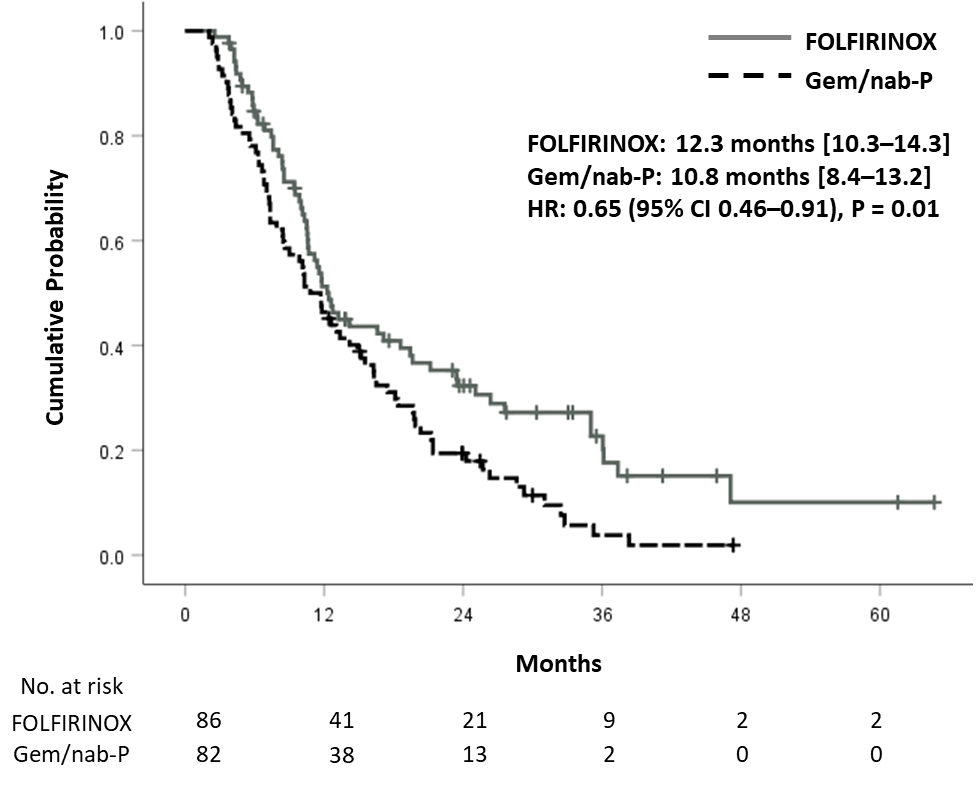 |
| --- | --- |

Supplementary Figure 3. Kaplan–Meier curves of overall survival among patients with ECOG 1+ in the (A) whole and (B) matched populations.

| A  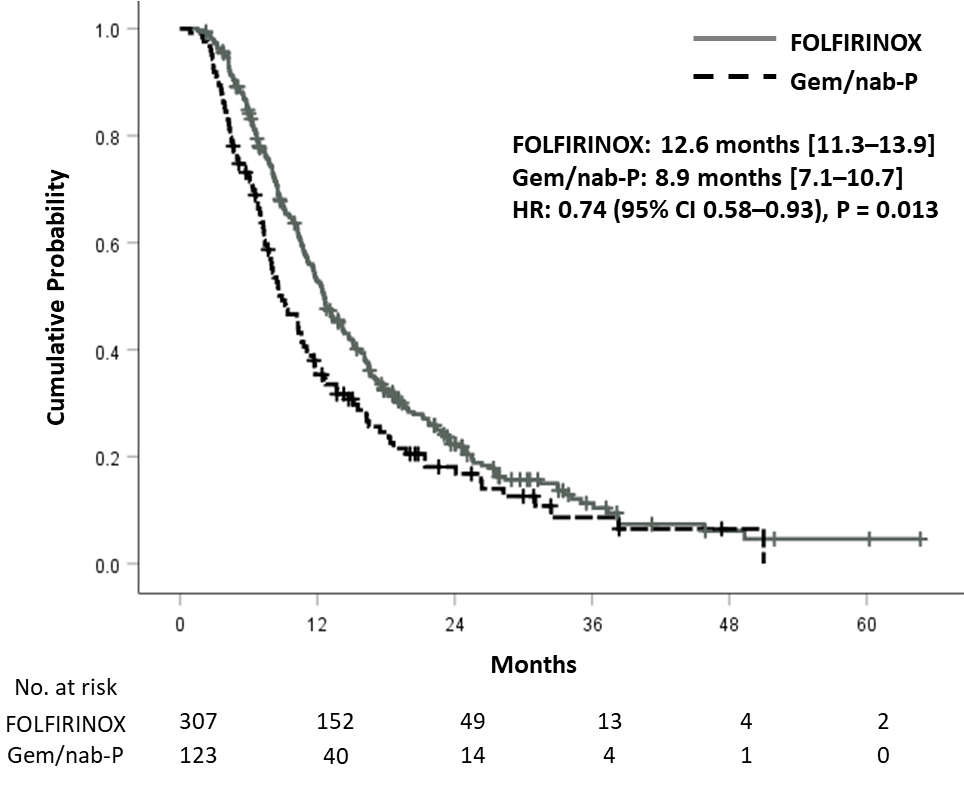 | B  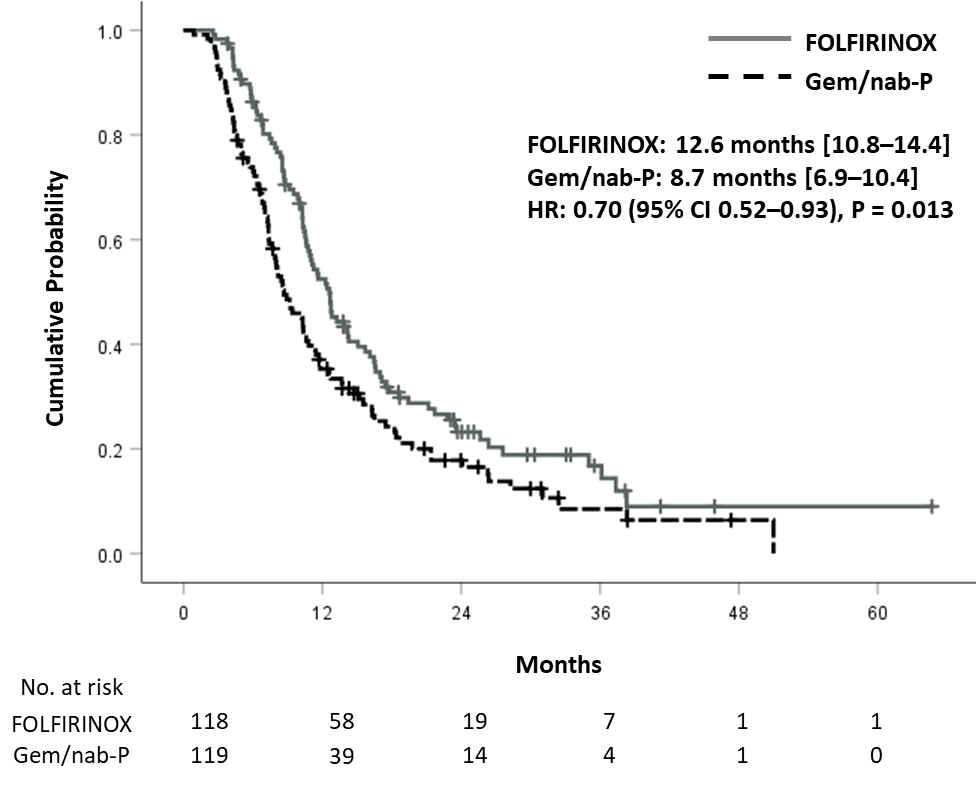 |
| --- | --- |

Supplementary Figure 4. Kaplan–Meier curves of overall survival among patients without previous pancreatic resection in the (A) whole and (B) matched populations.

| A  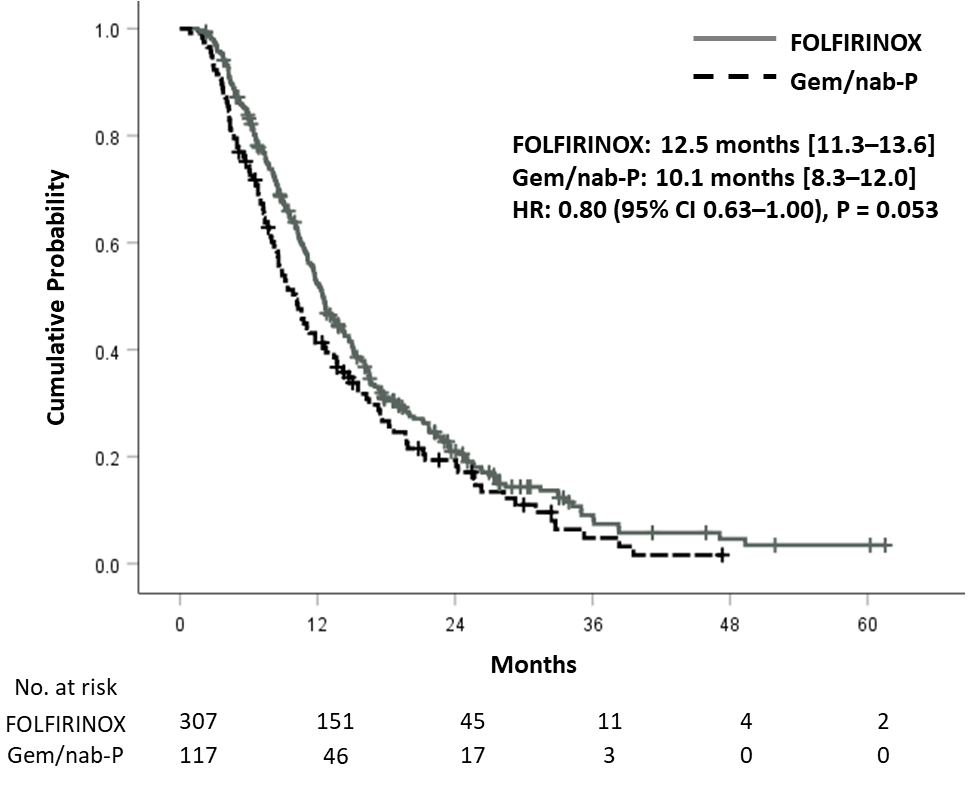 | B  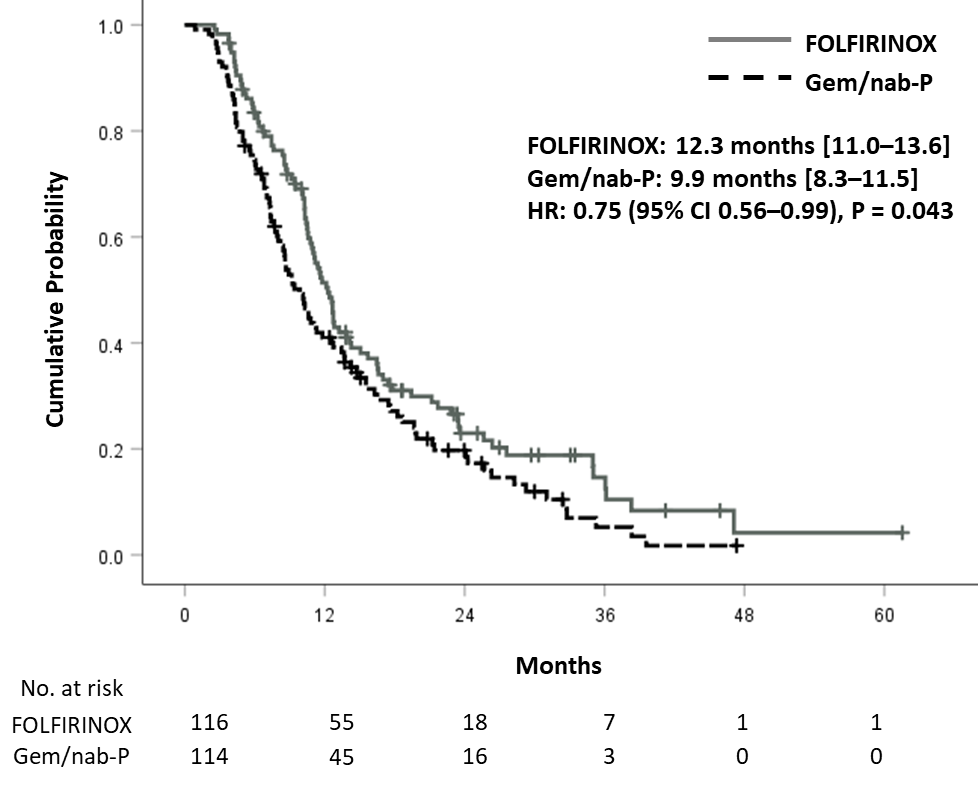 |
| --- | --- |

Supplementary Figure 5. Kaplan–Meier curves of overall survival in patients with sequential treatment according to patients’ age. (A) Age < 65 years; (B) Age ≥ 65 years.

| A  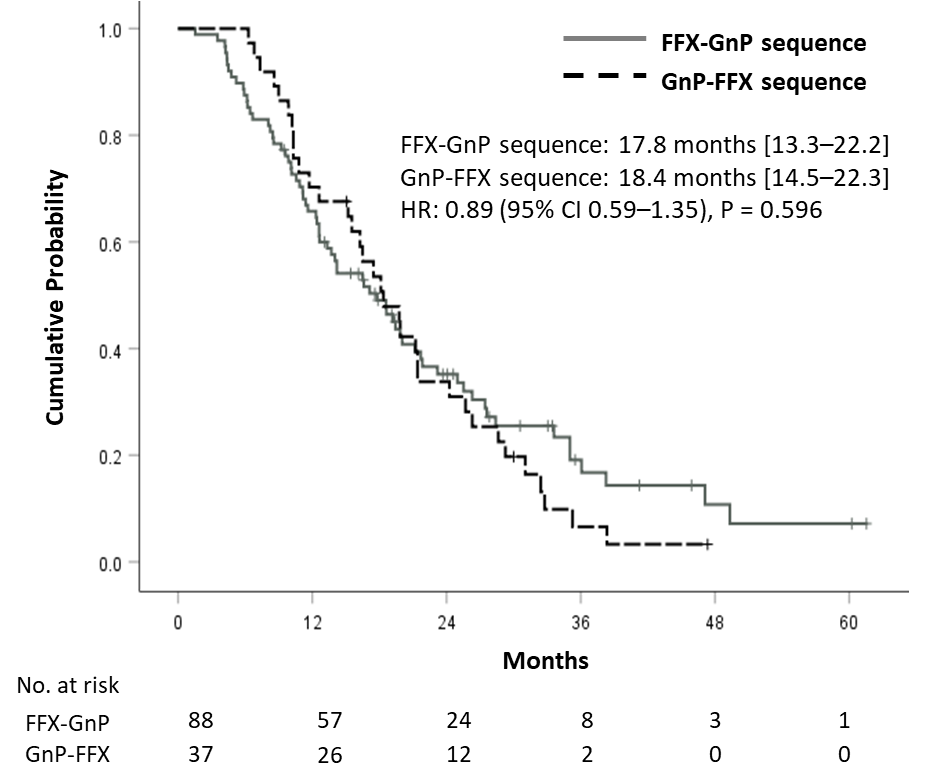 | B  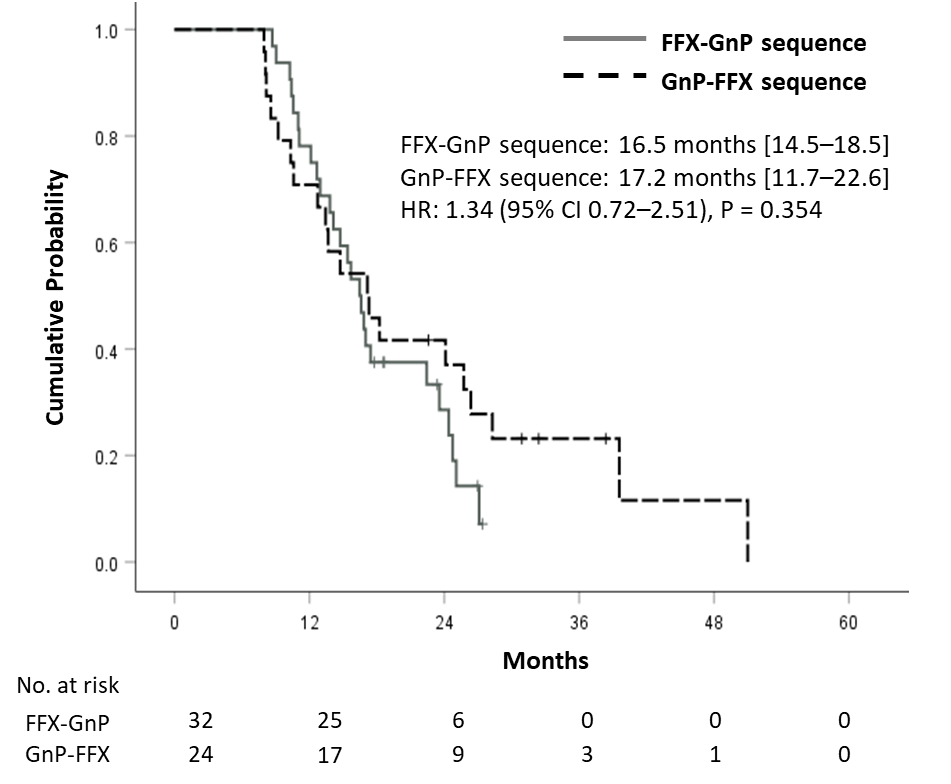 |
| --- | --- |

Supplementary Figure 6. Kaplan–Meier curves of overall survival in patients with sequential treatment according to performance status. (A) ECOG-PS 0; (B) ECOG-PS 1.

| A  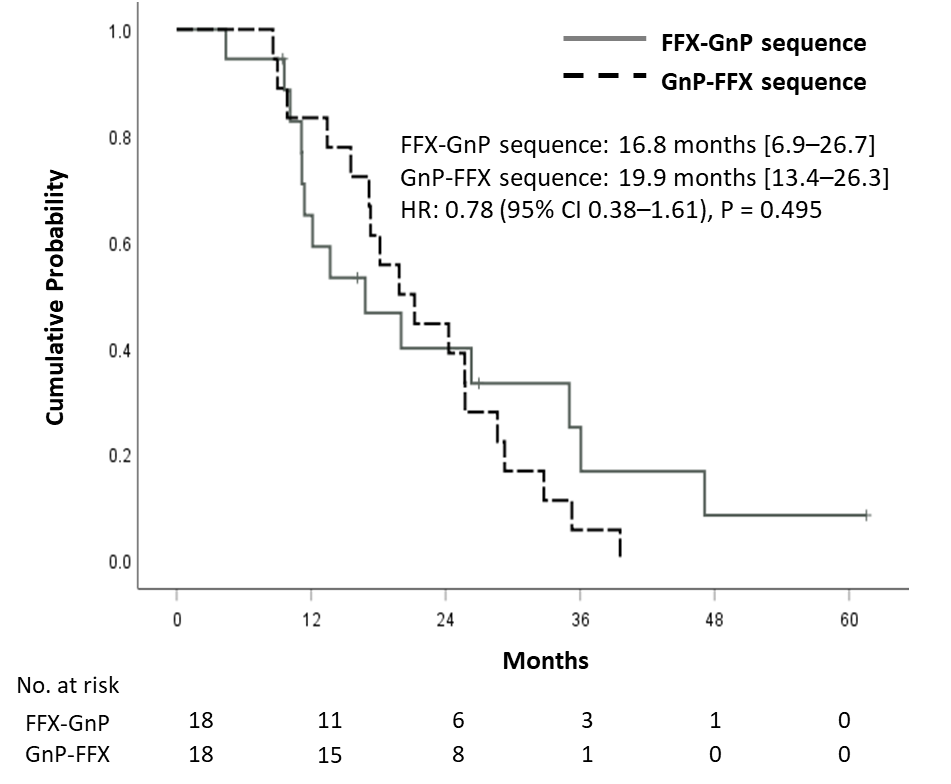 | B  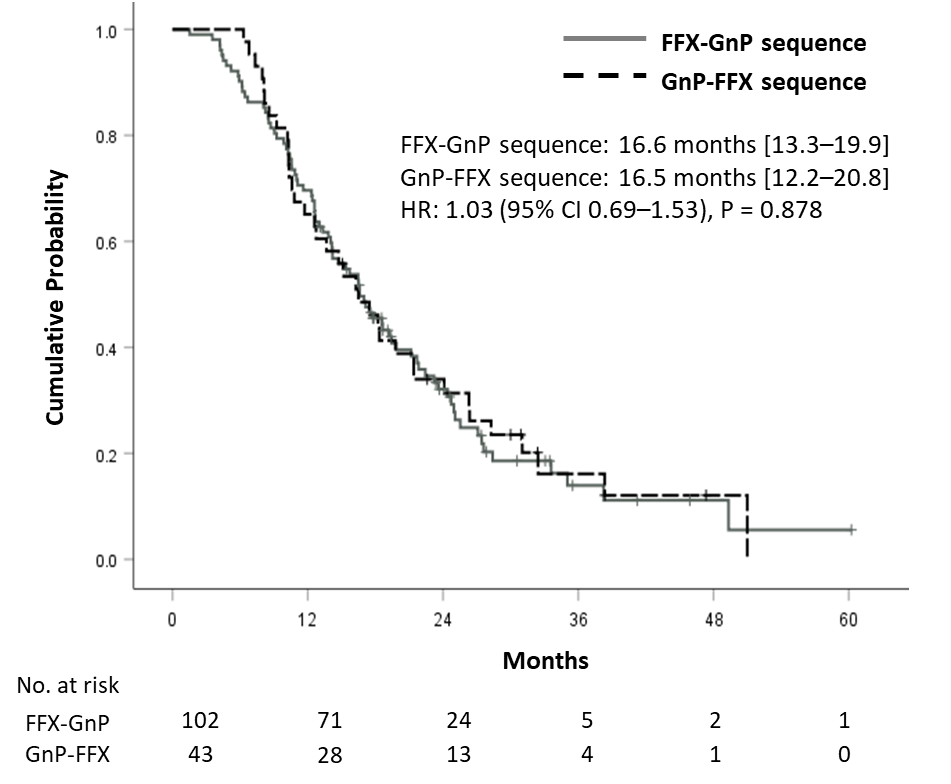 |
| --- | --- |

Supplementary Figure 7. Kaplan–Meier curves of overall survival in patients with sequential treatment according to history of pancreatic resection. (A) Naïve status; (B) With resection.

| A  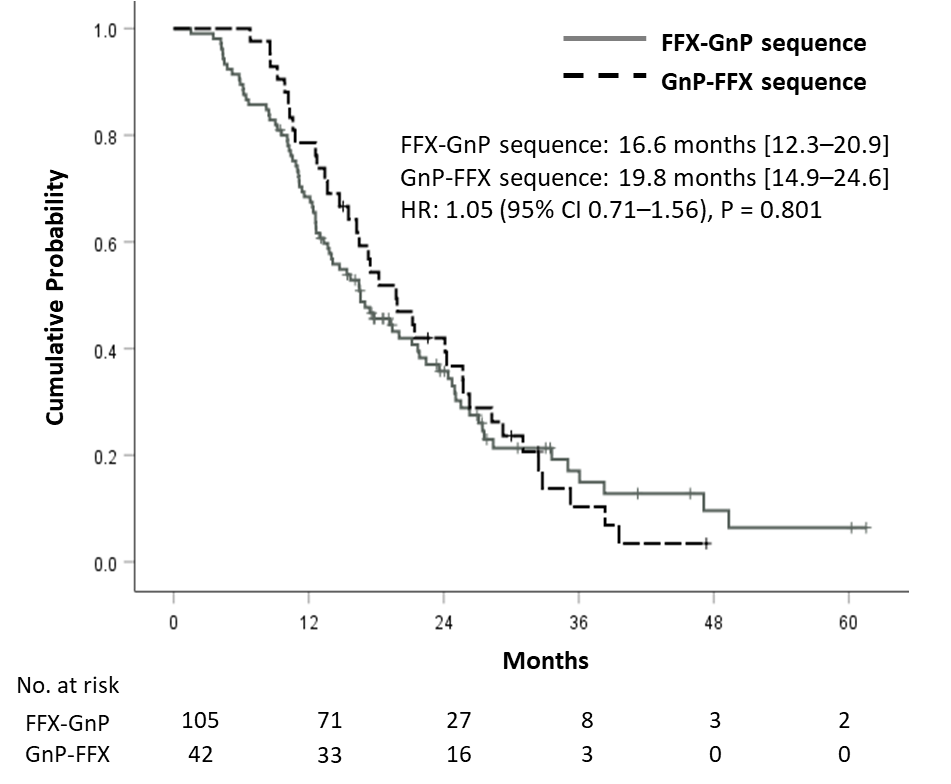 | B  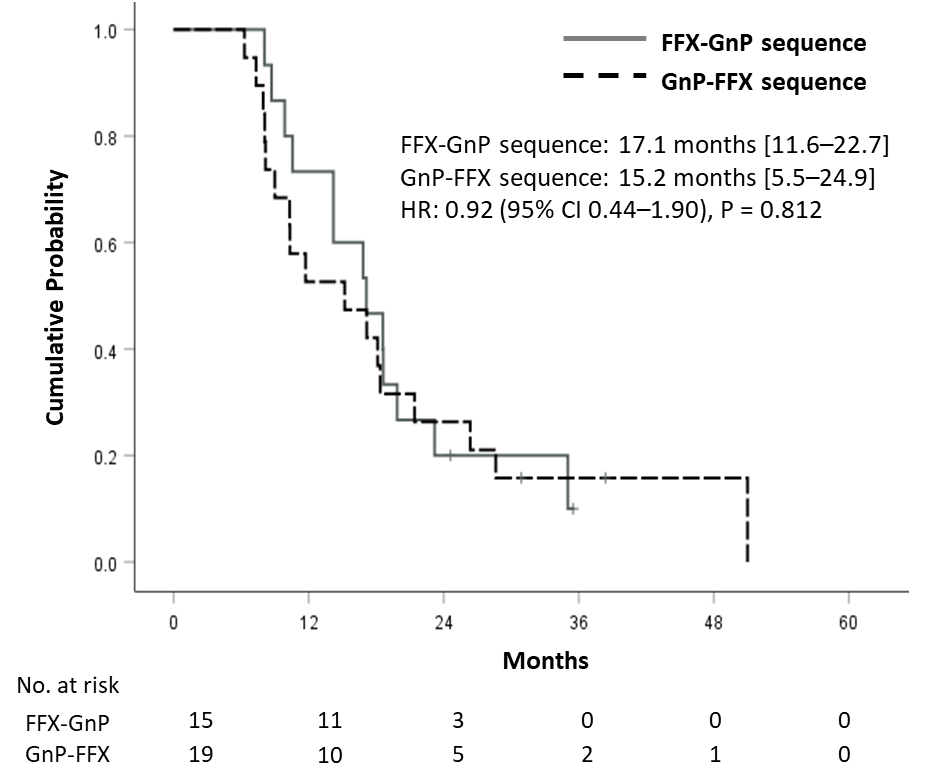 |
| --- | --- |

Supplementary Figure 8. Kaplan–Meier curves of overall survival in patients with interruptions of chemotherapy (A) before matching and (B) after matching.

| A  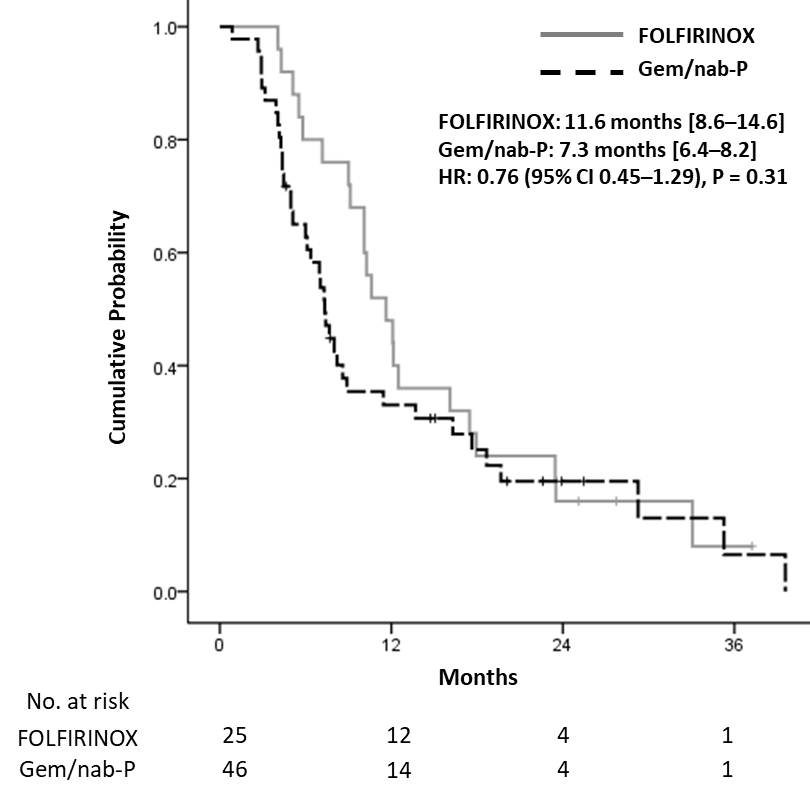 | B  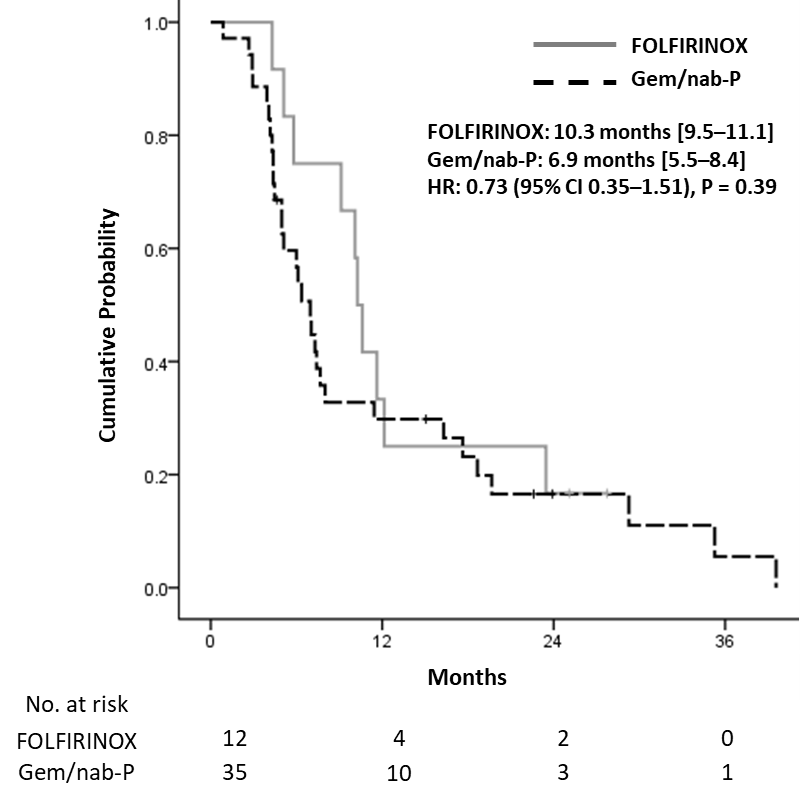 |
| --- | --- |

Supplementary Figure 9. Kaplan–Meier curves of overall survival in patients with cessation of chemotherapy (A) before matching and (B) after matching.

| A  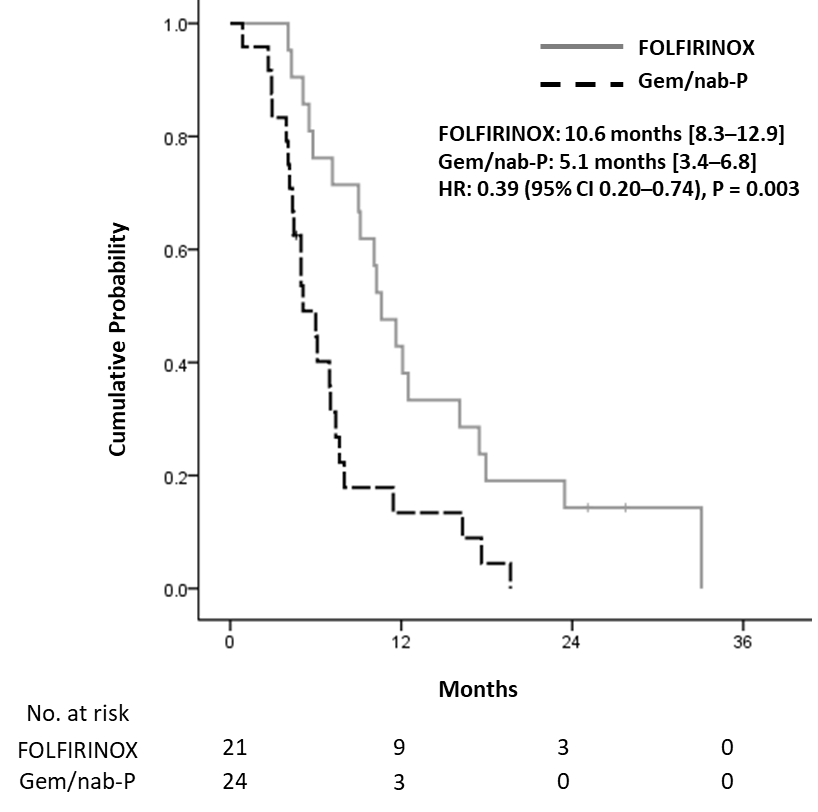 | B  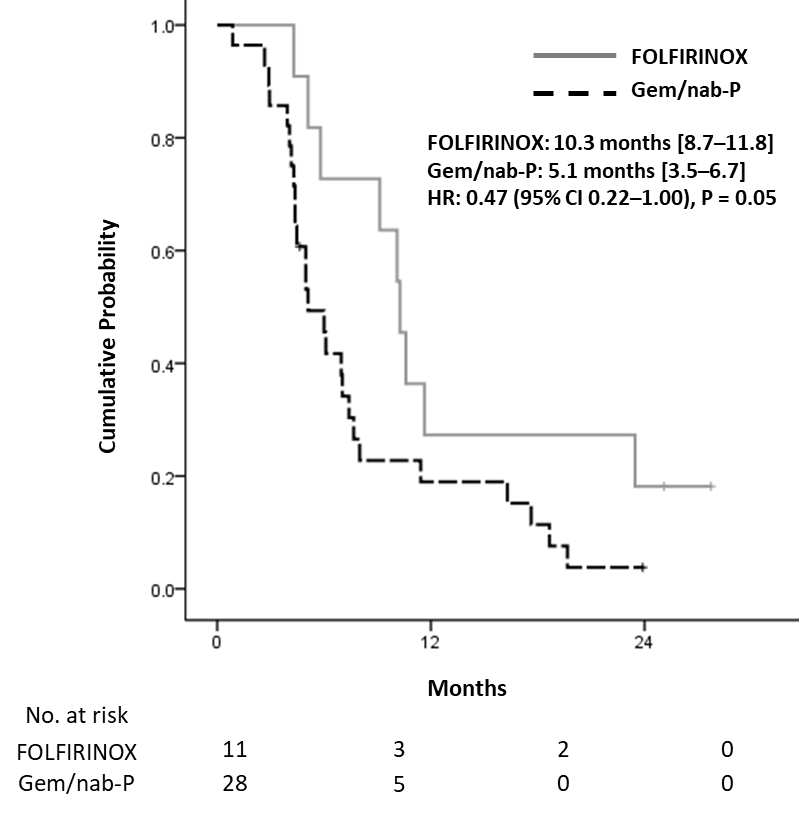 |
| --- | --- |
